# Fracture Resistance of Endodontically Treated Maxillary Premolars Restored by Various Direct Filling Materials: An In Vitro Study

**DOI:** 10.1155/2016/9138945

**Published:** 2016-08-30

**Authors:** Jozef Mincik, Daniel Urban, Silvia Timkova, Renata Urban

**Affiliations:** ^1^Private Dental Practice, Vystavby 3, 040 11 Kosice, Slovakia; ^2^Mint Dental, Private Dental Practice, Ostravska 8, 040 11 Kosice, Slovakia; ^3^Department of Dentistry and Maxillofacial Surgery, Faculty of Medicine, Pavol Jozef Safarik University, Rastislavova 43, 040 11 Kosice, Slovakia; ^4^First Department of Dentistry, Faculty of Medicine, Pavol Jozef Safarik University, Trieda SNP 1, 040 11 Kosice, Slovakia

## Abstract

The aim of this study is to compare the effect of various restorative materials on fracture resistance in maxillary premolars. Premolars (*n* = 64) with no restorations or cracks were selected. MOD cavities were prepared considering the buccolingual width to be equal to half of the intercuspal distance. The specimens were randomly divided into 8 groups, 8 specimens each:* group A* intact teeth,* group B* unfilled cavity,* group C* composite made by oblique layering technique,* group D* composite with 2 mm cusp coverage,* group E* bulk-filled posterior composite,* group F* glass-ionomer,* group G* amalgam, and* group H* composite with proximal boxes. The specimens were subjected to an axial compression load with the mean values of fracture resistance in group A: 1289 N, group B: 181.75 N, group C: 445.38 N, group D: 645.88 N, group E: 355.13 N, group F: 352.00 N, group G: 191.38 N, and group H: 572.00 N. There was no significant difference between groups B and G, between C and D, E, and F, and between group D and H. All other measurements were statistically significant. We conclude that composite restoration with cusp coverage is the most ideal nonprosthetic solution for endodontically treated teeth. Cusp coverage increases the fracture resistance compared to the conventional cavity design.

## 1. Introduction

Together with dental caries and periodontal disease, tooth fracture is the most common cause of tooth loss. Fractures are even more frequent in root-filled teeth. Excessive removal of coronal and radicular dentin during the root canal treatment (RCT) and lower residual moisture reduce the strength and increase tooth's fragility. Loss of axial walls, quite common in teeth that require RCT, also significantly weakens the hard dental tissues [1]. Study conducted by Joynt et al. [2] claims that occlusal cavity preparation may reduce mechanical resistance of the remaining dental tissues by 20%. A need for removal of the marginal ridges widens the cavity even further into the interproximal space. Resistance of dental structures reduces by 2.5-fold. This results in an overall 46% reduction in its mechanical resistance. In case when both marginal ridges are affected, the resistance decreases by 63%. Dalpino et al. [3] showed that mesio-occluso-distal (MOD) cavity design measuring half of the intercuspal distance, rounded internal angles, and either convergent or divergent angulation of internal walls weakens the remaining tooth structure. Relatively wide MOD cavities restored with amalgam frequently develop cusp fractures due to continuous functional occlusal forces. This is mainly caused by the inability of amalgam to strengthen weakened cusps [4]. Studies investigating this matter raised hope for recognizing the elements that can reinforce the remaining tooth structure. This can be accomplished by the application of adhesive restorative materials. However, some questions still require more evidence in order to provide conclusive answers. What restorative technique should be used to better reinforce the remaining tooth structure? How important is the role of adhesive agents that bond the restorative material to the tooth? Is it possible to reproduce the initial resistance of the tooth after the preparation? Ever improving quality of advanced dental materials and progress in manufacturing of bonding agents make the reinforcement of tooth structures possible [5]. The aim of our study is to compare the fracture resistance of endodontically treated maxillary premolars. These were restored by direct MOD fillings using various restorative materials. We investigated what impact might the cavity design (cusp preservation, cusp reduction, and proximal cavity), filling material (composite, glass-ionomer, and amalgam), and filling technique (incremental, bulk-fill) have on the overall mechanical resistance.

## 2. Materials and Methods

We selected a total of 64 human caries-free premolar teeth with no restorations or cracks. Buccolingual dimension of selected teeth varied between 8.12 and 10.03 mm. Each tooth was examined under a 10x magnification and those presenting visible enamel cracks or fractures were rejected. Teeth had been extracted within the previous 3 months for orthodontic reasons. Following the extraction, teeth were stored in 100 percent humidity throughout the study period. Maxillary premolars were selected since they are appropriate for evaluation of the efficacy of materials to increase their fracture resistance. Their anatomy, function, crown size, and crown/root ratio may make them more prone to fracture than other posterior teeth. Moreover, considering their location in the dental arch, they are subjected to both compressive and shear forces.

The premolars were embedded in an acrylic cylinder (external diameter 20.0 mm, height 20.0 mm) up to 2.0 mm below the cementoenamel junction (CEJ) using self-curing acrylic resin. MOD cavities were prepared using a high-speed handpiece and a diamond bur with continuous water cooling. Cavity preparation was initiated by an occlusal approach with a spherical diamond bur. Removal of the pulp chamber roof and reduction of mesial and distal walls were achieved with a cylindrical diamond bur, creating a 4 mm deep interproximally extended cavity. The buccolingual width of the isthmus was approximately one half of the intercuspal distance. Group D received a further 2.0 mm reduction of both buccal and palatal cusps. The cavity dimensions were carefully assessed with a digital caliper for proper standardization (Mitutoyo 500 173 Digimatic Caliper 300 mm/12 in, Mitutoyo Europe GmbH).

**Figure 1:**
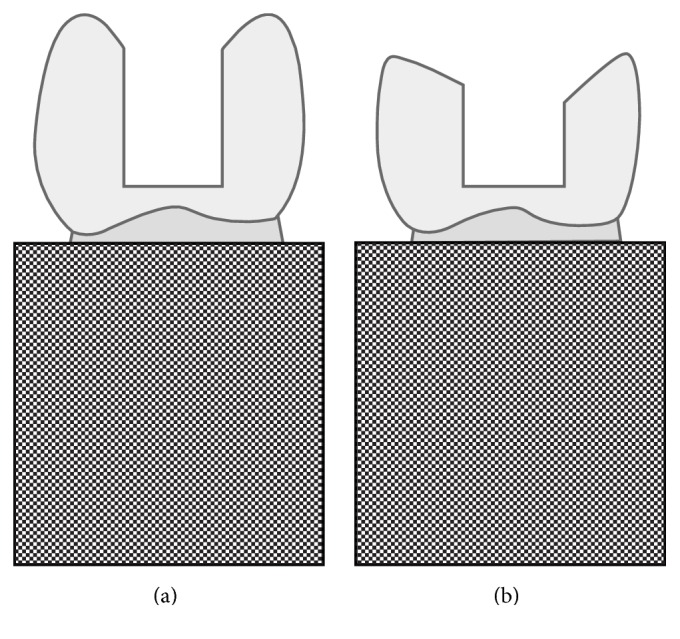
Specimens without (a) and with (b) cusp reduction as described by Lia Mondelli et al. [6].

The specimens were randomly divided into 8 groups with 8 teeth each:* group A* (control) included intact teeth,* group B* included teeth with unfilled MOD cavity,* group C* had teeth restored with nanohybrid composite (GrandioSO®, VOCO GmbH) using oblique layering technique,* group D* contained teeth restored with composite (GrandioSO, VOCO GmbH) with 2 mm cusp reduction (Figure 1),* group E* included teeth bulk-filled with hybrid posterior composite (X-tra fil®, VOCO GmbH),* group F* included premolars restored with fast setting glass-ionomer restorative (IonoStar Plus®, VOCO GmbH),* group G* had teeth restored with amalgam material (Tytin®, Kerr Corporation), and finally* group H* had teeth restored with composite (GrandioSO, VOCO GmbH) while the cavity was prepared to simulate the treatment of a vital tooth with mesial and distal boxes.

The floor of the exposed pulp chamber was treated with a layer of glass-ionomer cement. Preparation was completed and all surfaces were washed and air-dried using water and compressed air. Specimens in* groups C*,* D*,* E*, and* H* were conditioned with bonding agent (Futurabond M, VOCO), a single-component, light-curing, self-etching agent reinforced with nanoparticles.

Specimens in* group H* received the occlusal isthmus of 4 mm buccolingually with pulpal floor 2 mm deep. The buccolingual widths of mesial and distal boxes were 4 mm wide, comparable to the width of the occlusal isthmus. Each interproximal box had a gingival floor depth of 2 mm mesiodistally and axial wall height of 4 mm. Margins were prepared with 90° cavosurface angle.

## 3. Testing Conditions

**Figure 2:**
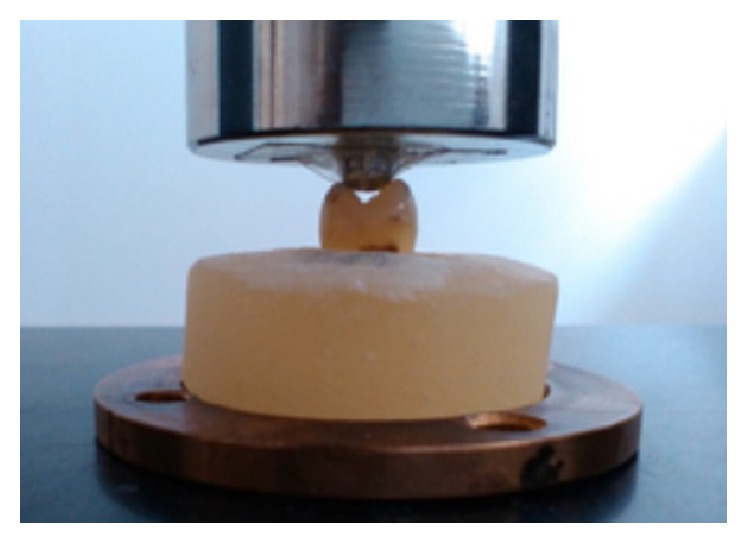
Mounted tooth in the acrylic cylinder under the LR5k testing machine.

After 48 hours of storage, the specimens were mounted in an universal testing machine (LR5k, Lloyd Instruments) and subjected to an axial compressive load applied parallel to the long axis of the tooth and to the slopes of the cusps. A steel sphere (4 mm wide) loaded the buccal and lingual cusps of the tested specimens at a crosshead speed of 0.5 mm/min, until fracture occurred. The load required to inflict fracture was expressed in N (newton) as registered by the machine (Figure 2).

Statistical analysis was performed using STATA/IC statistical software package (version 13.1 for Mac, StataCorp, USA).

## 4. Results

The measurements were analyzed by the Shapiro-Wilk test for normality (*P* > 0.05) and Kolmogorov-Smirnov test for equal distribution (*P* > 0.1). Data were normally and uniformly distributed; parametric tests were used for further analysis. Descriptive statistics provided the mean fracture resistance values for each group with the value of standard deviation (SD) and maximal and minimal values as shown in Table 1.

One-way analysis of variance was used to evaluate the significance of differences between groups at a level of difference of 0.0001 (Table 2).

The Tukey multiple comparisons test revealed significant differences among groups. Significant differences were found between the specimens of group D (cusp coverage) and others, except for group H. There was no significant difference between group C (oblique layering) specimens and both groups E (*bulk composite*) and F (*GI filling*). In addition, no statistically significant difference was found between group G (*amalgam*) and group B (*unfilled MOD cavity*). All other comparisons between the groups show statistically significant differences. Higher level of significance was present in comparison to amalgam fillings. The results of the measurements made between selected groups are shown in Table 3.

## 5. Discussion

Main reason for the selection of premolars in this study lies in their morphology. Cuspal inclination of premolars renders them more susceptible to cusp fracture under occlusal force. MOD cavities were designed in order to mimic a situation that may often be seen in clinical settings. Comparable situation has also been extensively reproduced in other clinical studies (Table 4). This in vitro study investigated the fracture resistance of premolars with weakened class 2 MOD cavities. Teeth were restored using different restorative materials. The most severe consequence of poor mechanical resistance is a cusp fracture. Since tooth fracture is a common occurrence in clinics, study of this pathology remains relevant. Several studies have examined the incidence of dental fractures. They have established that the incidence of fractures is more frequent in premolars [7, 8]. Other authors investigated the relation between various direct restorations and their ability to reinforce the MOD cavities in premolars after the root canal treatment (Table 4). The mechanical resistance of sound teeth in other studies with the values varying from 825 N [9] to 1514 N [6] is comparable to the values found in our measurements. When comparing resistance of unfilled MOD cavities, deviations presented in selected papers are greater: from 171 [10] to 565 [11]. This deviation may be explained by different means of cavity preparation and different testing equipment. The diameters of testing sticks vary from 4.0 to 8.0 mm. In our work the diameter was 4.0 mm which translates into relatively higher loading pressure to which the tested specimen is exposed. It is our opinion that the differences in absolute values may be adjusted using relative difference between the resistance of the unfilled cavity and restored cavities.

Our results show that the best means for increasing the mechanical resistance of the cavity is to include cusp coverage (capping). Cusp coverage may be a safe option for restoring teeth weakened by root canal treatment. The values of resistance in our study were significantly higher in comparison to the majority of studies with the exception of group H. Specimens in group H were prepared in order to mimic the preservation of tooth's vitality. Similar results were found in the works of Torabzadeh et al. [12], Lia Mondelli et al. [6], Xie et al. [13], and Panahandeh and Johar [14].

Amalgam restorations had the lowest fracture resistance. Mechanical resistance values of amalgam restoration in our study are on average only 10 N higher than that of an unfilled cavity. This corresponds with the fact that the amalgam has no adhesion to the cavity walls. In this case Shafiei et al. advocate the use of cuspal coverage with combined composite-amalgam in endodontically treated maxillary premolars [15]. Results of glass-ionomer restorations were very promising. The mechanical resistance of GIC is comparable and statistically insignificant in comparison to the composite fillings (GI mean 352 N, composite mean 445 N). However, it may be important to consider that the mechanical resistance of GIC decreases rapidly after two years. Thus the GIC may be recommended as a relatively long term temporary filling material. It can be indicated for MOD cavities in case when immediate permanent restoration is not feasible. Cavity design that utilizes the interproximal box preparation shows minimal improvement of mechanical resistance values compared to conventional MOD cavities. This may support the evidence claiming that marginal ridge conservation (tunnel preparation) could be in some instances more beneficial technique. Bulk-fill composites show similar resistance values compared to conventional composite resins (445 N and 352 N, resp.).

## 6. Conclusion

Our results show that a composite restoration with cusp coverage is in fact the most ideal nonprosthetic solution for endodontically treated teeth. Such restorations withstand occlusal forces that are 200.5 N stronger than restorations made by oblique layering technique and 454.5 N stronger than amalgam restorations. Buccal and lingual cusp coverage in extensive MOD cavities of maxillary premolars significantly increases the fracture resistance compared to the more conventional cavity design. Modern fast setting glass-ionomer cement with mechanical parameters similar to that of composites may be recommended as a long term temporary fillings. Clinical success is determined by the application of sound biomechanical principles adapted to the specific tooth and specific clinical situation.

## Figures and Tables

**Table 1:** The comparison of the average values for resistance between the groups.

*n* = 8	A	B	C	D	E	F	G	H
Mean	1289.00	181.75	445.38	645.88	355.13	352.00	191.38	572.00
SD	257.75	57.98	23.75	38.43	37.23	26.34	60.98	61.82
Min	972	113	405	584	300	326	124	475
Max	1700	267	468	688	404	404	277	617

*F*  (variance ratio) = 100,034798,  *P* < 0,0001

A (intact), B (unfilled), C (composite oblique layering), D (composite cusp coverage), E (bulk fill), F (glass-ionomer), G (amalgam), H (proximal box cavity).

**Table 2:** One-way analysis of variance of differences between groups.

Source of variation	Sum squares	DF	Mean square
Between groups	7130092.75	7	1018584.678571
Within groups	570209	56	10182.303571
Corrected total	7700301.75	63	

**Table 3:** Tukey multiple comparisons test between selected groups.

Group comparison	MD (95% CI)	L/SE (L)	Significance
B versus G	−9.625	0.269788	NS (*P* = 1.000)
C versus D	200.5	5.62	*HS (P* = 0.005)
C versus F	93.375	2.617294	NS (*P* = 0.589)
C versus E	90.25	2.529701	NS (*P* = 0.630)
D versus E	290.75	8.149701	*HS (P* < 0.001)
D versus H	73.875	2.070711	NS (*P* = 0.823)
E versus F	3.125	0.087594	NS (*P* = 1.000)

Mean difference (MD), confidence interval (CI), high significance (HS), no significance (NS), B (unfilled), C (composite oblique layering), D (composite cusp coverage), E (bulk fill), F (glass-ionomer), G (amalgam), group H (proximal box cavity).

**Table 4:** Summary of other studies testing the mechanical resistance on premolars.

Author (reference)	UP	UF	MOD	CC	CO	AMG
Javaheri et al. [16]	1139		919			705
Atiyah and Baban [17]	1123	545	687		672 SI	
Lia Mondelli et al. [6]	1514		605	1419		BX
Moosavi et al. [18]			803		754 BF	BX
Ragauska et al. [19]	1218		941		1407 IY	BX
Santos and Bezerra [20]	1138	490	1054			
Shafiei et al. [21]	1101	228		699		772 AC
Kikuti et al. [22]	940	460	780		520 SI	
Torabzadeh et al. [12]	1051		791	1148	800 OY	
Xie et al. [13]	1131		904	1085		
Yamada et al. [9]	825		700			
Shivanna and Gopeshetti [10]	1098	171	440		524 FRC	
Fahad and Majeed [23]	1182	556	879		855 BF	
Panahandeh and Johar [14]			873	1499	750 1CC	BX
Sharma et al. [24]	1193	248	867			501
Sarabi et al. [25]	1196			962		
Pradeep et al. [11]	1139	565	778			818
Joshi et al. [26]	1005	221	720		841 FRC	BX

Unprepared tooth (UP), unfilled cavity (UF), cusp coverage (CC), one cusp coverage (1CC), composite other (CO), bulk-fill (BF), Siloran (SI), inlay (IY), onlay (OY), box preparation (BX), fibre-reinforced composite (FRC), amalgam capping (AC).
